# Supplementary material for: Are carbon emissions trading and green financial instruments synergistic? -Comprehensive quantitative research based on content analysis
Source: PLoS One. 2024 Mar 7;19(3):e0298601. doi: 10.1371/journal.pone.0298601 (PMC10919601; doi:10.1371/journal.pone.0298601)
Supplement: S1 Data — (ZIP) [file pone.0298601.s001.zip › Data/地级市Z_F工作报告.zip!/╜¡╬≈/╕╙╓▌╩╨/└┤╫╘╣1⁄2╓┌║┼í╛┬φ┐╦╩2╛▌═°í┐_2017.docx]

各位代表：

现在，我代表市人民政府，向大会报告政府工作，请予审议，并请政协委员和列席会议的同志提出意见。

一、过去五年工作回顾

市四届人大一次会议以来，在省委、省政府和市委的坚强领导下，市人民政府团结依靠全市人民，深入贯彻落实中央和省、市委决策部署，抢抓机遇、砥砺奋进，实现了“十二五”圆满收官、“十三五”良好开局。

五年来，赣南苏区振兴发展卓有成效，综合实力显著增强。争取出台《国务院关于支持赣南等原中央苏区振兴发展的若干意见》，赣南苏区振兴发展上升为国家战略。国家层面支持赣南苏区政策文件125个。全面执行西部大开发政策，162项具体政策得到落实。对口支援工作成效明显。2012—2015年，上级下达赣州各类补助资金1375亿元，年均增长20.2%。获批全国加工贸易承接转移示范地等国家级重大平台8个，龙南、瑞金经开区和赣州高新区升格为国家级园区，赣州综合保税区、进境木材国检监管区建成运营，赣州铁路国际集装箱场站获批国家临时口岸对外永久开放，全国内陆口岸首个国检监管试验区挂牌成立，赣州成为全国拥有国家级平台最多的设区市之一。突出民生问题加快解决，完成农村危旧土坯房改造69.5万户、低电压改造54万户，解决300万农村人口饮水安全问题，新建改造农村公路1.4万公里、桥梁556座，新（改、扩）建校舍401万平方米，D级校舍危房全面消除。主要经济指标增速高于全国、全省平均水平，人均水平与全国、全省差距进一步缩小。地区生产总值接近2000亿元，年均增长10.9%。人均生产总值突破3000美元。财政总收入、一般公共预算收入、规模以上工业增加值、固定资产投资总额实现翻番。固定资产投资增速连续四年全省第一，农村居民人均可支配收入增速连续三年全省第一。《若干意见》提出的中期目标基本实现。

五年来，产业转型升级步伐加快，发展质效不断提高。工业加快发展。规模以上工业主营业务收入突破3000亿元，稀土钨、家具产业集群主营业务收入均突破千亿元，战略性新兴产业主营业务收入突破400亿元。中国南方稀土集团组建运营。新能源汽车科技城、“中国稀金谷”等一批项目启动。新增高新技术企业126家，2项科技成果获国家科技进步二等奖，科技进步贡献率55%。农业增产增效。建成高标准农田135万亩，粮食生产“十二连丰”。赣南脐橙品牌价值居全国初级农产品类价值榜榜首。赣南茶油获批国家地理标志保护产品。蔬菜、生猪等产业加快发展。赣县、信丰被认定为国家现代农业示范区。服务业量质齐升。金融业增加值占GDP 比重5.2%。旅游接待总人数、总收入年均分别增长28%和32.6%。瑞金列为国家历史文化名城，共和国摇篮景区晋升国家5A级景区。赣州获批粤赣皖苏四省物流大通道节点城市。电商交易额连续三年翻番。服务业增加值占GDP比重提高4.2个百分点。

五年来，基础设施明显改善，城乡面貌深刻变化。全市常住人口城镇化率45.5%，提高近8个百分点。南康撤市设区，中心城区建成区面积141平方公里，人口141万。赣县撤县设区获批。蓉江新区启动建设。赣南大道、南门口下穿隧道及黄金、沙石、武陵、客家四座大桥建成通车，完成文清路、厚德路、健康路商业街改造提升，灶儿巷、郁孤台—八境台历史文化街区整治修缮。黄金机场改扩建加快推进。赣韶铁路、赣龙铁路复线建成通车，昌赣客专开工建设，赣深客专、兴泉铁路正式获批，铁路运营总里程达555公里。瑞寻、隘瑞、赣崇、龙杨、昌宁、寻全等6条高速公路建成通车，实现县县通高速，高速公路总里程达1116公里。升级改造国省道2884公里，实现县县通国道。赣州500千伏电网与江西主电网形成双通道，220千伏变电站实现县县全覆盖，全市供电能力实现翻番。4Ｇ网络覆盖所有城区、乡镇和80%以上行政村。植树造林337万亩，森林覆盖率稳定在76.2%以上。“河长制”启动实施。县县建成城镇生活污水处理厂。节能减排规划目标全面完成。中心城区环境空气质量优良率90%以上，全市重要水功能区水质达标率96.5%，城市集中式饮用水源地水质达标率100%。荣获全国首批生态文明典范城市。

五年来，改革开放不断深化，内生动力更加强劲。部省共建教育改革发展试验区、城乡建设用地增减挂钩、农民住房财产权抵押贷款、“救急难”等68项省级以上试点扎实推进。农村土地承包经营权确权登记颁证基本完成。颁发全国第一本林权类不动产权证。企业注册资本登记、“三证合一、一照一码”①等商事制度改革全面实施。金融改革创新取得突破，在全国首创险资支农支小模式，组建金盛源担保公司、金融资产交易中心，设立全国第一家私募基金呼叫中心，全市各类金融机构167家，赣州成为周边四省九市金融机构聚集度领先、门类最齐全的城市。成功争取建立东江流域上下游横向生态补偿机制，组建全省首家环境能源交易所，成立林业产权交易中心。国资国企、教育文化、医疗卫生、社会治理等重点领域和关键环节改革扎实推进。央企、民企入赣成果丰硕，“赣商回归、赣人兴赣”工程扎实推进，引进省外资金、实际利用外资年均分别增长20.8%、11.4%，外贸企业由不到800家增加到2000余家，出口总额实现翻番。

五年来，民生民计持续改善，发展成果普惠共享。民生类财政支出超过1300亿元，年均增长24%。脱贫攻坚富有成效，减少贫困人口145.2万人，贫困发生率下降20.6个百分点。五大社会保险扩面提标。城乡低保、农村五保户集中分散供养、企业离退休人员养老金、失业保险金月均发放标准累计增幅均达50%以上。累计新增城镇就业50万人，城镇登记失业率控制在3.3%以内。完成各类棚户区改造7.4万户，建设保障性住房11.6万套，解决66.4万中低收入群众住房困难问题，住房保障面积提高到人均15平方米以上。学前教育快速发展，义务教育普及成果有效巩固，高中阶段教育质量稳步提高。赣南教育学院升格为赣州师范高等专科学校，赣南卫生健康职业学院获批设立，赣州职教园区启动建设。县级公立医院综合改革全面推开。市人民医院新院投入使用。开通中心城区至龙南、瑞金、上犹、崇义4条城际公交快线。“菜篮子”工程加快实施。公共文化服务标准化建设成效明显，城乡文化惠民工程扎实推进。全民健身活动深入开展，成功承办第十四届省运会、举办第四届市运会。安全生产和食品药品安全形势稳中向好。平安赣州、法治赣州建设深入推进，获评全国法治宣传教育先进城市，连获三届“全国双拥模范城”、综治“长安杯”，蝉联五届“全国综治工作优秀市”。

五年来，政府自身建设得到加强，行政效能进一步提升。扎实开展政府系统党的群众路线教育实践活动、“三严三实”专题教育和“两学一做”学习教育，认真落实中央八项规定精神和省市若干规定，政府作风进一步好转。法治政府建设深入推进。新一轮政府机构改革和职能调整顺利完成。绩效管理考核全面实施。“放管服”改革不断深化，市本级行政审批事项精简为122项，非行政许可审批事项全部精简到位。“六单一网”②建设扎实推进。自觉接受市人大法律监督和市政协民主监督，建议和提案办结率、满意和基本满意率均为100%。强化监督执纪问责，政府廉政建设不断加强。

各位代表！奋斗充满艰辛，成绩来之不易。这主要得益于党中央、国务院的深切关怀，国家部委的倾情支援；得益于省委、省政府和市委的正确领导，市人大、市政协和社会各界的监督支持；得益于各级各部门和全市人民的团结奋斗、顽强拼搏。在此，我代表市人民政府，向全市广大干部群众，向各位人大代表、政协委员和离退休老同志，向各民主党派、工商联、无党派、人民团体和社会各界人士，向驻市解放军指战员、武警官兵，向所有关心支持赣州发展的海内外朋友，表示崇高的敬意和衷心的感谢！

回顾五年奋斗历程，我们深切体会到，办好赣州的事情，必须坚持解放思想不停步，以新理念引领发展行动，不断向改革开放要动力，向创新创业要活力，向特色优势要竞争力；必须咬住发展不放松，以“跳起来摘桃子”的勇气担当，苦干实干，打好六大攻坚战，实现经济社会发展新跨越；必须坚定绿色发展不偏移，不以牺牲环境为代价换取一时的经济增长，坚持走可持续发展之路；必须致力改善民生不动摇，把群众的安危冷暖时刻放在心上、扛在肩上，让赣南人民有更多的获得感和幸福感。

我们也清醒地认识到，赣州经济社会发展还面临不少困难和问题。主要是：经济总量、均量仍然偏小；工业基础薄弱，服务业规模较小，产业结构不优，创新能力不强；城乡发展不平衡，基本公共服务水平较低，脱贫攻坚任务依然艰巨；政府法治建设、作风建设、廉政建设需进一步加强，等等。对此，我们将采取有力措施，切实加以解决。

二、今后五年目标思路

今后五年，是实施“十三五”规划、实现同步全面小康的决胜期，也是大有作为的重要战略机遇期。尽管宏观经济形势复杂多变，但我国经济发展长期向好的基本面没有变。《若干意见》政策红利加速释放，全省“龙头昂起、两翼齐飞、苏区振兴、绿色崛起”发展格局进一步提升，赣州的战略地位和后发优势日益突显。市第五次党代会绘就的宏伟发展蓝图，六大攻坚战激发的干事创业热情，汇聚了全市上下共谋发展、齐促振兴、同奔小康的强大正能量。我们要因势而谋、因势而动、因势而进，推动赣州经济社会发展年年都有新变化、三年迈上新台阶、五年实现新跨越！

今后五年工作的总体思路是：高举中国特色社会主义伟大旗帜，以邓小平理论、“三个代表”重要思想、科学发展观为指导，深入学习贯彻习近平总书记系列重要讲话精神，协调推进“四个全面”战略布局，牢固树立五大发展理念，深入实施《若干意见》，解放思想，内外兼修，北上南下，坚决打好主攻工业、精准扶贫、新型城镇化、现代农业、现代服务业、基础设施建设六大攻坚战，统筹抓好改革开放、生态文明、社会民生等各项工作，着力加强政府自身建设，加快赣南苏区振兴发展，实现与全国同步全面建成小康社会。

今后五年，全市经济社会发展的总体目标是：

——综合经济实力实现新跃升。经济保持中高速增长，地区生产总值、财政总收入年均增长10%左右，地区生产总值突破3000亿元，财政总收入突破500亿元，人均主要经济指标与全国、全省平均水平的差距明显缩小，成为全省最具潜力、支撑江西协调发展的重要增长板块。

——发展协调性显著增强。工业实现“三年翻番”③，到2020年，规模以上工业主营业务收入达8000亿元，工业固定资产投资达1500亿元，服务业增加值超1500亿元。产业结构进一步优化，现代产业体系基本建立，科技进步贡献率提高到60%。统筹城乡发展加快推进，城乡基础设施更加完善，区域性现代化中心城市基本形成。

——改革开放取得新突破。先行先试成效显著，重要领域和关键环节改革取得决定性成果，若干领域改革走在全国、全省前列。对内对外开放广度和深度不断拓展，新型内陆口岸基本建成，成为内陆开放型经济新高地。

——生态优势充分彰显。资源节约集约利用率不断提高，可持续产业结构、生产方式和消费模式基本形成，单位生产总值能耗明显下降，城市空气质量持续改善，城镇生活污水集中处理率、城镇生活垃圾无害化处理率提高到85%，森林覆盖率稳定提升，我国南方地区重要的生态屏障基本建成。

——人民生活更加幸福美好。城镇居民人均可支配收入年均增长10%，农村居民人均可支配收入年均增长11%，城乡居民收入差距进一步缩小。脱贫攻坚任务三年完成。覆盖城乡的社会保障体系更加完善，社会事业更加繁荣，基本公共服务水平接近或达到全国平均水平。民主法治建设和精神文明建设取得新进步。社会治理体系和治理能力现代化取得新进展，人民群众安全感和幸福指数明显提升。

各位代表！目标引领未来，实干成就梦想。只要我们团结一心、众志成城、接力奋斗，振兴发展时代荣光一定会闪耀赣南大地，同步全面小康梦想一定会变为美好现实！

三、今后五年工作重点

深入推进赣南苏区振兴发展战略，举全市之力打好六大攻坚战，狠抓项目带动，集聚发展新动能，厚植发展新优势。

（一）突出打好主攻工业攻坚战

加快构建特色鲜明、集约高效、环境友好、市场竞争力强的新型工业体系，以工业崛起撑起振兴发展脊梁。

打造三大增长板块。依托赣州都市核心区建设，重点发展稀土钨新材料及应用、新能源汽车及配套、生物制药、家具、高端装备等产业，打造中部核心增长板块；依托瑞兴于经济振兴试验区建设，带动周边县重点发展现代轻纺、食品药品、氟盐化工、新型建材等产业，打造东部增长板块；依托“三南”加工贸易重点承接地建设，推动相关区域紧密协作，重点发展电子信息、稀土钨深加工、食品加工等产业，打造南部增长板块。支持各工业园区发展首位产业，加快完善园区基础设施、配套设施和公共服务平台，推动园区向产城融合新区转型，把园区建成宜业宜居的绿色工业新城。

加快产业集群发展。建设新能源汽车科技城，打造国内重要的新能源汽车产业基地。建设“中国稀金谷”，打造国内知名的稀土稀有金属高新技术产业集聚区。建设电子信息产业科技城，承接珠三角电子信息产业转移，打造“赣粤高铁”沿线电子信息产业带。建设“青峰药谷”，打造具有国内领先水平的集药材种植加工、药品研发制造、医药健康旅游为一体的产业基地。建设现代家居城，推动家具产业向智能化、定制化、生态化升级。改造提升传统优势产业，促进纺织服装、食品加工等产业向品牌化、个性化转型。推动“互联网+”协同制造，加快培育发展新一代信息技术、生物制药、节能环保、新能源等新兴产业。培育形成稀土新材料及应用、钨新材料及应用、新能源汽车及配套、电子信息、铜铝有色金属、以家具为主的现代轻纺等6个主营业务收入超千亿元的主导产业，形成生物制药、氟盐化工和新型建材等3个主营业务收入超200亿元的产业集群，形成若干个主营业务收入超100亿元的新兴产业集群。

帮扶企业发展壮大。实施重点企业培育工程，打造中国南方稀土集团、青峰药业等一批主营业务收入过百亿元的“企业航母”，培育100户主营业务收入超10亿元的龙头企业、100户境内外上市或新三板挂牌企业。加大对“专精特新”中小微企业支持力度，创建小型微型创业创新示范基地，大力扶持创新型、成长型企业发展。实施质量强市战略，培育一批拥有自主知识产权、具有核心竞争力的知名品牌和优势企业。加快培育成千上万扎根赣州的本土企业，形成“大企业顶天立地、小企业铺天盖地”的生动局面。

（二）决战决胜精准扶贫攻坚战

把脱贫攻坚作为重大政治任务，立下愚公志，啃下硬骨头，确保2018年现行标准下贫困人口全面脱贫、贫困县全部摘帽。

打好脱贫攻坚组合拳。落实产业扶贫“五个一”机制④，因地制宜发展特色扶贫产业，充分吸纳贫困户参与产业链脱贫。统筹易地搬迁、农村危房改造、农村保障房建设等政策，改善贫困家庭居住条件，三年完成易地搬迁扶贫15万人。实施健康扶贫工程，筑牢居民基本医疗保险（新农合）、大病保险、疾病医疗商业补充保险、医疗救助等“四道保障线”，坚决遏制因病致贫、因病返贫。优先支持贫困村教育发展，阻断贫困代际传递。统筹就业、金融、旅游、电商、生态、光伏等多种有效扶贫模式，探索创新社会扶贫方式。实施社会保障政策性兜底脱贫工程，推进扶贫政策与低保政策有效衔接，实现应保尽保、应扶尽扶，不让一个贫困户在全面小康路上掉队！

推进整村扶贫开发。整合资源，精准投入，全方位扶持贫困村发展，实现932个贫困村旧貌换新颜。推进“一村一品”建设，每个贫困村培育1个以上龙头企业或合作社。统筹推进贫困村基础设施建设，深入开展危旧空心房整治、生活垃圾和污水治理。积极争取央企、大型民企结对帮扶贫困村，设立产业投资基金、扶贫公益基金，支持贫困村发展产业、改善生产生活条件。完善村民活动室、卫生计生室、农家书屋及体育健身场所功能，确保2020年贫困村基本公共服务主要领域指标接近全市平均水平。

完善扶贫工作机制。健全市、县、乡、村四级扶贫责任体系，完善单位驻村扶贫、干部结对帮扶全覆盖工作机制，全面压实脱贫攻坚责任。构建完善社会扶贫信息服务平台，实现社会扶贫资源精准化配置。健全扶贫开发投入稳定增长机制，支持贫困县开展统筹整合使用财政涉农资金试点。强化扶贫政策、项目、资金专项审计巡视。完善贫困对象精准识别、动态管理、精准退出、减贫脱贫考核等机制，对低收入人群实施滚动帮扶，确保脱贫不返贫。

（三）精心组织新型城镇化攻坚战

构建以中心城市为龙头，瑞金、龙南次中心城市和周边卫星城市为依托，县城和特色中心镇为支点的赣南城镇体系，加快推进以人为核心的新型城镇化。

做大做强中心城市。建成赣州西站交通枢纽、迎宾大道—文明大道快速路、赣南大道快速路、东江源快速路、武陵大道、蓉江三路、红旗大道东延、客家大道西延、赣储路等项目，实施城区快速公交工程，推进蓉江新区跨江桥梁、红旗大桥、南河大桥改造、全球通大桥改造、水西穿赣江至水东隧道等项目建设，研究启动城市地铁建设，加快构建以“四横六纵一环”快速路网⑤、“八纵十一横”主干路网⑥、城市轨道交通为主的现代都市交通体系。高标准建设蓉江新区，打造现代化新城和新型产业聚集区。推进赣州经开区产城融合发展，建设高端产业聚集区。加速南康、赣县融入主城区步伐，建设产业升级与承接转移示范区、高新技术产业基地。完善提升河套老城区，繁荣章江新区，焕发赣州历史名城和现代新城魅力。

提升城市建管水平。实施欧潭湿地公园、生态林地公园、蓉江新区中心公园、狮形岭湿地公园、三江口地区环境景观改造等项目，推动公园广场、居住小区升级改造，完善市政和公共服务设施，打造“10分钟便民生活圈”。树立“精明增长、紧凑城市”理念，推进空间规划编制和“多规合一”，划定保护范围和建设控制带，降低容积率和建筑密度，增加绿地面积。优化城市设计，注重控制和引导城市空间形态、景观视廊、公共空间和建筑风格，塑造赣州城市个性特色。推动绿色建筑、装配式建筑发展，提升建筑质量和品质。统筹推进城市排水防涝设施、地下综合管廊和海绵城市建设。改革城市管理执法体制，建设和完善数字城管指挥平台。深入开展市容环境综合整治，严厉打击违法违章建设。鼓励企业和市民积极参与城市建设和管理，实现城市共治共管、共建共享。

促进城乡统筹发展。做强做优县城，提升县城规模等级、集聚能力、承载功能和宜居环境，建设人口集中、产业集聚、土地集约的“小县大城”。推进龙南撤县设市，把龙南建成赣深高铁沿线工业强、旅游旺、生态美的开放型城市。推进瑞金市省直管县体制改革试点，把瑞金建成宜居宜业宜游、更具活力实力魅力的赣闽边际区域性中心城市。统筹推进户籍管理、土地管理、社会保障等配套改革，加快农业转移人口市民化。以示范镇、中心镇为重点，打造一批特色工业强镇、农业大镇、商贸重镇、文化名镇、旅游旺镇。完善村庄规划体系，规范农民建房管理，加快完善基础设施，推进人居环境整治，提升公共服务功能，加强传统村落和历史文化名镇名村保护，建设和谐秀美新农村，让农村成为望山见水忆乡愁的好去处。

（四）深入实施现代农业攻坚战

推动一二三产业融合发展，加快农业大市向农业强市转变，努力实现农业强、农民富、农村美。

优化农业产业结构。实施“藏粮于地、藏粮于技”战略，提高粮食综合生产能力。实施脐橙产业发展升级行动计划，建设中国供销·赣南脐橙交易中心，建成具有国际影响的优质脐橙产业基地。推进油茶“三个中心、三个基地”⑦建设，提升赣南茶油品牌影响力，形成年产值超百亿元的油茶产业。打造105、323国道沿线蔬菜产业带，建成供深港重要蔬菜基地，打造南方重要蔬菜集散地。稳定提升生猪产业，加快发展牛羊规模养殖，推动家禽、水产、白莲、烟叶、茶叶、毛竹、刺葡萄、甜叶菊、花卉苗木等区域特色产业发展。

推进农业接二连三。实施农业产业集群培育工程，发展壮大集群核心企业，规划建设现代农产品加工区，培育销售收入100亿元以上产业集群3个，销售收入过50亿元的农产品加工园区3个，主要农产品加工率达60%以上，建成全国重要的特色农产品、有机食品生产加工基地。重点围绕畜禽养殖、园艺生产、大田种植等，建设一批农业物联网技术应用示范点。大力发展农村电子商务，拓展农村二三产业增收空间。大力发展休闲观光、分享、定制、创意、养生农业，让农业成为有奔头的产业。

强化农业支撑保障。坚持最严格的耕地保护制度，全面划定永久基本农田。大力开展土地整治，加快高标准农田建设，推进农业机械化。完善乡镇农业服务中心管理运行机制，推广优势特色产业关键技术。推进“百县百园”建设，每个县（市、区）建成1个初具规模、产业融合度高、示范带动作用强的现代农业示范园。加快发展智慧农业，推进农业信息进村入户。加强农产品质量安全监管能力建设，建立主要农产品质量安全追溯体系。推进农业适度规模经营，加快农村土地有序流转，培育新型经营和服务主体，构建完善现代农业产业体系、生产体系和经营体系。

（五）全力推进现代服务业攻坚战

推动现代服务业提质增效，建成赣粤闽湘四省边际区域性金融、物流、电商、文化旅游和健康养老中心，打造全国服务业发展示范基地。

培育壮大现代金融业。规划建设金融商务核心区、金融虚拟产业园和金融后援服务中心。大力引进各类金融机构，发展地方法人金融机构。推动要素市场建设，充分利用资本市场扶贫政策，推动企业上市挂牌、并购重组、发行债券。发展各类基金、互联网金融、金融中介机构和金融外包产业，建设赣州金融后台中心。推进大数据和区块链技术运用，完善社会信用体系建设。开展大宗农林产品、特色农业保险、农业巨灾保险等新业务，完善农村担保服务体系，推进城乡小额贷款保证保险试点，实现政府融资担保机构县域全覆盖。

加快发展文化旅游业。围绕建设“一核三区”⑧，大力发展全域旅游，推动旅游产业转型升级。建成中心城区“三江六岸”水上旅游观光带等一批龙头项目。积极创建国家5A、4A级旅游景区，国家级、省级旅游度假区和生态旅游示范区。发展提升红色旅游、休闲度假旅游和乡村旅游，打造周边四省九市自驾游集散中心。深入挖掘赣州特色文化资源，推动文旅融合发展，创新开发旅游演艺、旅游体验、旅游参与等旅游产品，打响红色故都、江南宋城、客家摇篮、绿色家园、两江源头等旅游品牌，建成全国著名的红色旅游目的地、东南沿海地区休闲度假后花园。

集聚发展现代物流、电商等服务业。创建国家现代物流创新发展、技术应用试点城市，推进国家级物流园区、货运枢纽等试点项目建设。建立全市统筹的区域电商平台和物流快递信息化平台，打造稀有金属、家具、脐橙和跨境电商交易平台，建成一批国家级、省级电商示范基地和电商产业集聚区。积极培育研发设计、信息技术、服务外包等新业态。建设医养结合示范城市，大力发展旅居、生态、休闲、温泉、观光等产业，打造国内知名的养生养老示范基地。

（六）全面展开基础设施建设攻坚战

坚持统筹兼顾、合理布局、适度超前，强力推进基础设施建设，加快构建功能完善、安全高效的现代化基础设施体系，全面提升振兴发展的支撑保障能力。

完善综合交通网络。建成昌赣客专、赣深客专、兴泉铁路，建设长赣铁路、瑞梅铁路，争取开工建设赣郴永兴铁路、南丰至瑞金城际铁路、赣韶铁路扩能改造等项目，构建“一纵一横”高速铁路网⑨和“两纵两横”普速铁路网⑩，基本实现县县通铁路。建成兴赣、宁安、安定等高速公路，建设广吉高速宁都段、大广高速南康南互通、兴赣高速北延、大广高速复线等项目，构建“三纵三横六联”高速公路网⑪，形成通达长珠闽“4小时高速经济圈”。提升水运通航能力，实现赣州至南昌三级通航。完成黄金机场改扩建，开通国际航线，新建瑞金支线机场，启动龙南、宁都通用机场前期工作，构建通达港澳台和国内枢纽城市的航空网络，建成全国重要的区域性综合交通枢纽。

提升能源保障和信息网络水平。推进华能瑞金电厂二期、信丰火电等电源点建设。实施特高压电网建设，加快赣州西500千伏输变电等主电网、100千伏及以下配电网、农村电网升级改造工程建设。完善油气管网对接覆盖，推进省天然气管网（赣州段）工程建设，实现长输管道天然气利用全覆盖。推进“宽带中国”示范城市建设，实施“光网城市”、“无线城市”、“宽带乡村”工程，加快智慧城市建设，规划建设云计算大数据中心和公共信息资源数据库，构建高速、移动、安全、泛在的新一代信息基础设施。

加快水利基础设施建设。推进284个城镇防洪工程建设，确保中心城区和县城以及重点乡镇达到国家规定防洪标准。开展病险山塘、水库、水闸、圩堤除险加固，基本消除水利工程安全隐患。加强主要支流和中小河流重要河段治理。新建一批中小型水库和抗旱应急水源工程，完成19个城市应急水源工程、上犹江饮水工程一期暨龙华水厂建设。加强农田水利基础设施建设，持续开展大、中小型灌区续建配套与节水改造，实施节水灌溉增效示范项目、“五小”水利工程、小水电增效扩容技改项目，提高水利设施保障能力。

（七）着力深化改革开放

加速释放改革红利，提升对外开放水平，努力在新一轮改革开放中走在前列。

深化重点领域改革。深化商事制度改革，加强事中事后监管，推进信用监督、警示和惩戒机制建设。深化农村集体产权制度改革，构建完善农村产权交易平台，加大确权赋能力度，不断激发农村活力。深化财税体制改革，实施跨年度预算平衡机制和中期财政规划管理，扩大财政专项资金竞争性分配改革试点。深化投融资体制改革，大力推行政府和社会资本合作（PPP）模式，激活民间投资。深化价格体制机制改革，促进资本、技术、土地、人力资源等要素市场发展。深化国资国企改革，健全国资监管体制，发展混合所有制经济，推进国有企业市场化战略重组，做大做强做优国有企业。深化扩权强县改革，促进县域经济发展。充分发挥对口支援优势，争取国家部委更多改革试点布局赣州。

激发创新创业活力。实施创新驱动“1122”工程⑫，建设创新型赣州。实施重大科技专项行动计划，重点围绕稀土钨新材料、新能源汽车、生物制药、电子信息等战略性新兴产业，加快关键核心技术突破和产品创新，加速创新成果转化和产业化。强化企业创新主体地位，支持企业与高校、科研院所组建产业创新联盟，构建产学研用协同创新体系。实施人才强市战略，坚持自主培养和外部引进并举，集聚一批产业创新领军人才。实施知识产权入园强企工程，推进重点产业专利导航⑬。鼓励支持科技创新人员、大学生、返乡农民工等重点人群创新创业，创建一批众创空间、创业咖啡、创新工场，让赣州成为实现梦想的创新乐土、创业家园。

打造内陆开放高地。全面对接长珠闽等沿海发达地区，建设赣粤、赣闽产业合作区。积极“南下”招商引资，突出驻点招商、专业招商、以商招商，推进“引资、引技、引智”三位一体招商。深入实施“赣商回归、赣人兴赣”工程，引进一批总部型、旗舰型大企业、大集团。推进黄金机场设立航空口岸，申报建设龙南保税物流中心，建设瑞金口岸作业区，完善赣州铁路国际集装箱场站功能，不断拓展多式联运通道。以设立国家临时对外开放口岸、国家级国检监管试验区为契机，规划建设临港经济区。努力把赣州建成“一带一路”的重要支点，内陆地区物流成本最低、产业服务体系最优的双向开放高地。

（八）持续加强生态文明建设

推动绿色发展，建设生态家园，争当全省生态文明试验区建设排头兵，打造美丽中国“赣州样板”。

加强生态环境保护和治理。严格落实生态保护管控红线，提升区域生态屏障功能和可持续发展能力。实施大气污染防治行动计划，加快重点行业脱硫、脱硝和除尘改造，积极推行清洁生产。实施低质低效林改造工程，10年改造完成1000万亩。实施水、土壤污染整治行动计划，加强水土资源保护和水土流失综合治理，加快废弃矿山复垦复绿，推进重点流域、重点区域重金属和水土污染防治。加强城镇和工业园区污水处理设施及配套管网建设，完成蓉江新区、水东片区等污水管网和污水处理设施项目建设。加强农村环境连片整治和农业面源污染防控。全面完成主要污染物减排任务。积极创建国家园林城市、国家森林城市、全省生态文明县（乡、村），建设绿色机关、学校、社区、企业和家庭，让保护生态环境成为全社会的自觉行动。

发展绿色生态产业。加快生态旅游、生态农业、林下经济、节能环保等绿色产业发展。深入实施绿色农业“十大行动”⑭，开发培育富硒农产品，让赣南农产品成为绿色、有机、健康、安全的代名词。加快绿色矿业发展示范区建设，提高稀土、钨等矿产资源综合利用率。实施重点领域节能降耗工程，加快淘汰落后和过剩产能。大力发展太阳能、风能、生物质能、地热能等可再生能源产业。推行企业、园区、产业循环化发展，构建绿色循环低碳的现代产业体系，为子孙后代留下可持续发展的“绿色银行”。

健全生态文明制度。健全完善源头严防、过程控制、损害赔偿、责任追究的生态文明制度体系。建立领导干部任期生态文明建设问责制，实行生态环境损害责任终身追究、领导干部自然资源资产和环境责任离任审计制度。全面推行“河长制”，建立水陆共治、部门联治、全民群治的河湖保护管理长效机制。实施东江源、赣江源生态补偿试点。创新和完善公众参与环境监督保护机制，推进环境污染第三方治理。用最严格的制度保护生态环境，让赣南大地青山常在、绿水长流、蓝天常驻！

（九）不断增进民生福祉

顺应人民群众新关切新期盼，加快补齐民生短板，努力提高人民群众对就业就医、教育文化、居住环境等方面的满意度。

保障和改善民生。坚持新增财力向民生领域倾斜，每年办好一批民生实事。实施保障性住房工程，推进公租房货币化补贴，2018年基本解决城市棚户区改造问题。完善覆盖城乡的公共就业服务体系，实现城镇新增就业40万人，城镇登记失业率控制在4%左右。优化城市公交，加强城市停车场、充电桩建设和管理，提升农村公路建设水平，扩大农村客运，让群众出行更便捷、更经济、更安全。健全覆盖城乡居民的社会保障体系，建立统一规范的社会保障服务中心，以基本养老、基本医疗、最低生活保障为重点，推动社会保障扩面提标、城乡一体。建立机关企事业单位职工工资正常增长机制，健全农民工工资支付保障机制。加大收入分配调节力度，提高居民转移性收入，增加低收入劳动者收入，让老百姓的钱包一天天鼓起来。

提升公共服务水平。推进城乡公办幼儿园建设，基本实现行政村及城区每5万人口有1所公办幼儿园。推进义务教育学校标准化建设，化解城区中小学校大班额问题，保障农民工子女平等入学，实现县域义务教育均衡发展。促进普通高中特色办学、多样化发展。每个县（市、区）建有1所特殊教育学校。建成赣州职教园区、赣南职业技术学院。落实乡村教师支持计划，培养一批名师和名校长。推进教育改革发展试验区建设，确保教育发展主要指标达到全国平均水平。实施医疗卫生、公共卫生、中医药服务体系建设工程，建成市妇幼保健院新院、市中医院新院、市儿童医院，支持人口大县建设三级综合医院，努力建成区域性医疗卫生中心，主要医疗卫生资源指标、居民主要健康指标达到全国平均水平。广泛开展群众性精神文明创建活动，争创全国文明城市。推进国家公共文化服务体系示范区、客家文化（赣南）生态保护实验区建设，建成市文化艺术中心，振兴赣南采茶戏，培育宋城文化、理学文化、阳明文化品牌，让赣南文化声名远播、发扬光大。深入开展全民健身活动，完善公共体育服务体系。坚持计划生育基本国策，优化出生人口结构和素质。完善养老服务体系、托底性社会救助体系，加强农村留守儿童关爱保护工作，促进慈善事业发展，以更完善的公共服务满足人民群众更高品质生活的需要。

深化社会治理创新。加强平安赣州、法治赣州建设，全面实施“七五”普法规划。健全落实社会治安综合治理领导责任制，完善立体化社会治安防控体系，严密防范、依法惩治各类违法犯罪活动，让老百姓白天出门放心、晚上睡觉安心。创新社会治理和服务机制，推进综治“三项建设”⑮，推行城乡社区网格化管理，加强重点、特殊人群监管和服务。依法规范改进信访工作，推行阳光信访、责任信访、法治信访。完善安全生产责任和监督管理制度，强化重点领域安全专项治理，坚决遏制重特大安全事故发生。加强国防动员和后备力量建设，深入开展双拥共建。健全应急管理体制机制，加强防灾减灾救灾体系建设。创建国家食品安全城市，以“零容忍”态度加强食品药品监管，切实维护人民群众饮食用药安全。

四、加强政府自身建设

把抓落实作为一切工作的生命线，大力弘扬苏区精神，转变工作作风，争创新时期“第一等工作”，努力建设人民满意政府。

坚持依法行政。全面正确履行政府职能，坚持法定职责必须为、法无授权不可为，做到有权不能任性、行权必须依法、用权必受监督，确保政府工作全面纳入法治轨道。完善政府立法体制机制，做好重点领域政府立法工作，提高政府立法公众参与度。坚持科学、民主、依法决策，对涉及群众切身利益的重大决策，及时组织专家论证、群众听证、风险评估和合法性审查。深化行政执法体制改革，推进执法重心下移，推行综合执法，健全行政执法和刑事司法衔接机制，提高行政执法公信力。自觉接受人大法律监督、政协民主监督和社会舆论监督，全面推进政务公开，实现公共权力、公共资源、公共服务公开透明，让权力在阳光下运行。

坚持高效施政。完善政府向社会力量购买公共服务机制，扩大政府购买服务范围，让群众享有更多更好的公共服务。综合运用大数据等手段，推进“互联网+政务服务”，实现全市政务信息系统互联互通、公共数据充分共享，打造“智慧政务”，提高行政效能。推进网上办事大厅建设，设立“政务超市”、“中介超市”。深化“放管服”改革，巩固提升“六单一网”成果，进一步减程序、减费用、减时限，打造全省乃至全国审批程序最少、办事效率最高、服务质量最好的发展环境。

坚持勤勉务政。始终保持干事创业的热情、雷厉风行的作风、勇于担当的精神，为赣州发展尽职、为全市人民尽责。加强学习型政府建设，强化公务员教育培训，提高公务员队伍专业化水平，提升抓落实的理解力、操作力和执行力。坚持讲实话、干实事，少发文、发短文，少开会、开短会，立说立行、马上就办、办就办好。开展常态化明察暗访，加强作风效能监督，对懒政怠政行为公开曝光、严肃追责。建立容错纠错机制，为勇于担当者担当，为改革创新者撑腰。

坚持廉洁从政。坚持把纪律挺在前面，加强政府廉政建设，扎紧制度笼子，筑牢思想防线，坚守廉政底线。严格落实中央八项规定精神、国务院“约法三章”，压紧压实“两个责任”和“一岗双责”，持续深入反对“四风”。健全惩治和预防腐败体系，围绕政府投资项目资金使用、工程招标投标、土地出让开发、政府采购和国有资产转让等重点领域，全面加强行政监察、审计监督和财政监督，深入查处发生在群众身边的腐败问题和不正之风，营造风清气正的干事创业环境。

各位代表！站在新的起点，我们比历史上任何时期都更接近赣南苏区振兴发展、同步全面建成小康的目标，比历史上任何时期都更有信心、有能力实现这个目标。让我们紧密团结在以习近平同志为核心的党中央周围，在省委、省政府和市委的坚强领导下，解放思想、真抓实干、开拓奋进，为创造更加美好的未来、让赣南老区人民过上富裕幸福的生活而努力奋斗！
